# Fine Structure of Plasmodesmata-Associated Membrane Bodies Formed by Viral Movement Protein

**DOI:** 10.3390/plants12244100

**Published:** 2023-12-07

**Authors:** Anastasia K. Atabekova, Sergei A. Golyshev, Alexander A. Lezzhov, Boris I. Skulachev, Andrey V. Moiseenko, Daria M. Yastrebova, Nadezda V. Andrianova, Ilya D. Solovyev, Alexander P. Savitsky, Sergey Y. Morozov, Andrey G. Solovyev

**Affiliations:** 1 A. N. Belozersky Institute of Physico-Chemical Biology, Moscow State University, 119992 Moscow, Russia; asya_atabekova@mail.ru (A.K.A.); sergei.a.golyshev@gmail.com (S.A.G.); lezzhov-genetic@mail.ru (A.A.L.); morozov@genebee.msu.ru (S.Y.M.); 2 Biological Faculty, Moscow State University, 119234 Moscow, Russia; bskulachev@gmail.com (B.I.S.); postmoiseenko@gmail.com (A.V.M.); 3 Faculty of Bioengineering and Bioinformatics, Moscow State University, 119234 Moscow, Russia; yastrebova_daria@mail.ru; 4 A. N. Bach Institute of Biochemistry, Research Center of Biotechnology of the Russian Academy of Sciences, 119071 Moscow, Russiaapsavitsky@inbi.ras.ru (A.P.S.); 5 All-Russia Research Institute of Agricultural Biotechnology, 127550 Moscow, Russia

**Keywords:** plant virus, endoplasmic reticulum, membrane contact, viral membrane compartment, Higrevirus, *Hibiscus green spot virus*

## Abstract

Cell-to-cell transport of plant viruses through plasmodesmata (PD) requires viral movement proteins (MPs) often associated with cell membranes. The genome of the *Hibiscus green spot virus* encodes two MPs, BMB1 and BMB2, which enable virus cell-to-cell transport. BMB2 is known to localize to PD-associated membrane bodies (PAMBs), which are derived from the endoplasmic reticulum (ER) structures, and to direct BMB1 to PAMBs. This paper reports the fine structure of PAMBs. Immunogold labeling confirms the previously observed localization of BMB1 and BMB2 to PAMBs. EM tomography data show that the ER-derived structures in PAMBs are mostly cisterns interconnected by numerous intermembrane contacts that likely stabilize PAMBs. These contacts predominantly involve the rims of the cisterns rather than their flat surfaces. Using FRET-FLIM (Förster resonance energy transfer between fluorophores detected by fluorescence-lifetime imaging microscopy) and chemical cross-linking, BMB2 is shown to self-interact and form high-molecular-weight complexes. As BMB2 has been shown to have an affinity for highly curved membranes at cisternal rims, the interaction of BMB2 molecules located at rims of adjacent cisterns is suggested to be involved in the formation of intermembrane contacts in PAMBs.

## 1. Introduction

Virus infection of plant tissues occurs by cell-to-cell transport of the viral genome through plasmodesmata (PD), an active process that requires virus-encoded movement proteins (MPs) [1,2]. In certain viruses, MP molecules form tubules that displace internal PD structures and serve as conduits for the transport of spherical or bacilliform virions into the cytoplasm of neighboring cells [3,4,5,6,7,8,9,10]. In the majority of plant viruses, however, MPs act in a more intricate manner, inducing only a transient modification of the PD structure [1,11,12]. The functions of the latter type of MPs include interaction with the viral genomic nucleic acid or virion resulting in the formation of a transport-competent entity, targeting of this genome transport form to PD, modification of the PD structure leading to increased permeability of the PD channels, and translocation of the viral genome through the modified PD into neighboring cells [1,11,12]. The exact molecular details of these steps of viral cell-to-cell transport are largely unresolved.

The function of MPs typically requires their interaction with cell endomembranes, in particular with the endoplasmic reticulum (ER). Indeed, the *Tobacco mosaic virus* (TMV) MP, which exemplifies a plant virus transport system with a single MP, has been shown to be associated with the ER [13,14,15,16]. A model has been proposed in which MP molecules form rafts in the ER membrane, which can then be transported via diffusion within the ER to PD. This provides a mechanism for the directed intracellular delivery of the transport form of the viral genome, which acts as a raft-interacting cargo [17]. Moreover, some ER-residing proteins, as well as proteins of ER–plasma membrane contact sites, can be directly involved in virus cell-to-cell transport [18,19,20,21]. Many groups of viruses encode transport systems that consist of two or more MPs, at least one of which interacts with the ER membranes [22]. One of these transport systems is the “triple gene block” (TGB), which encodes three MPs termed TGB1, TGB2, and TGB3; the TGB2 and TGB3 are the integral ER proteins [22]. Another such system is the “binary movement block” (BMB) encoding two MPs, one of which shows a marginal similarity to TGB2, integrates into the ER membranes, and modifies their structure [23,24].

Advances in the study of plant virus infections in recent decades have led to the understanding that viral genome replication and virus cell-to-cell transport are intimately linked [11]. It should be noted that viral replication occurs in association with cell membranes in specialized structures called “virus factories” or virus replication compartments (VRCs). The formation of VRCs requires virus-encoded proteins, namely the virus replicase, or MPs, or both [11]. For example, in potyviruses, the formation of VRCs, which represent vesicular structures detached from the ER membranes and containing virus replicase and genomic RNA, depends on the MP called 6K_2_, a small transmembrane protein [25,26,27,28]. In *Red clover necrotic mosaic virus* (RCNMV; genus *Dianthovirus*, family *Tombusviridae*), VRCs are formed by viral replicase components that induce reorganization and proliferation of the ER membranes. The RCNMV MP, which does not interact with membranes on its own, is recruited to these structures through an interaction with a cell protein that binds to one of the replicase components. Recruitment of the MP to VRCs is essential for efficient virus cell-to-cell transport [29,30,31]. 

The link between the replication and transport has been investigated in more detail for *Potato virus X* (PVX), a TGB-encoding virus. PVX replication occurs in VRCs associated with the PD orifices, suggesting a direct targeting of nascent genomic RNA progeny to the PD channels [32]. The PD-associated PVX-specific VRCs represent modified tubules of the cortical ER, described as “stacked membrane hoops” containing the PVX TGB2 protein [32]. In later stages of infection, a subset of PVX VRCs develops into an X-body: a relatively large perinuclear replication compartment that is thought to be organized similarly to PD-associated VRCs [33,34]. Analysis of the structure of X-bodies by super-resolution light microscopy has revealed that in these structures, the tubular ER is reorganized into densely stacked fine membrane hoops [33]. Interestingly, the PVX TGB1 protein, rather than the membrane proteins TGB2 and TGB3, is the major viral product that organizes the X-body [34]. Collectively, the PVX VRC data demonstrate that PVX TGB proteins induce reorganization of the cortical ER tubules to form the discrete replication/movement membrane compartments. According to studies of PVX protein interactions in plant cells, the TGB2 protein recruits both TGB3 and the viral replicase to the movement-related VRCs [35], whereas recruitment of TGB1 requires both TGB2 and TGB3 [32]. These data suggest that TGB2 may play a central role in the organization of PD-associated VRCs [36].

The genome of *Hibiscus green spot virus* (HGSV), which infects citrus trees [37], encodes BMB, a distinct transport system consisting of BMB1 and BMB2 proteins [23]. The BMB2 protein is located in PD-associated membrane bodies (PAMBs) that represent derivatives of the ER membranes [23]. As shown by inhibitory analyses, the mechanism of BMB2 intracellular transport to PAMBs is based on a lateral translocation in the plane of the ER membranes rather than on a secretory pathway [38]. In plant cells, BMB2 interacts with BMB1 and directs BMB1 to PAMBs, the PD interior, and neighboring cells [23,39]. The function of BMB2 in virus cell-to-cell transport depends on its ability to increase the permeability of the PD channels [24]. Being a membrane protein with two hydrophobic domains, BMB2 is shown to adopt a W-shaped topology in the ER membrane and to induce, upon its overexpression, constrictions of the ER tubules [24]. These properties of BMB2 are similar to those of reticulons, cell proteins that generate curvature of the lipid bilayer and thus shape the ER tubules [40,41,42,43]. The reticulon-like activity of BMB2 is correlated with its ability to increase the PD permeability [24]. The ability of BMB2 to induce constrictions of the ER tubules is hypothesized to be responsible for the formation of PAMBs [24]. As the PVX TGB protein, similar to BMB2, is able to induce ER tubule constriction [24], the BMB-specific PAMBs may be similar, both structurally and functionally, to the PVX PD-associated VRCs; however, the structure of PAMBs remained so far unknown.

In this paper, the fine structure of HGSV BMB2-specific PAMBs was analyzed using AiryScan confocal microscopy, transmission electron microscopy, and electron tomography. Further, BMB1 and BMB2 proteins were localized in PAMBs by use of immunogold labeling, and interaction between BMB2 molecules in PAMBs was demonstrated by Förster resonance energy transfer between fluorophores detected by fluorescence-lifetime imaging microscopy.

## 2. Results

### 2.1. Visualization of PAMBs by AiryScan Confocal Microscopy

**Figure 1:**
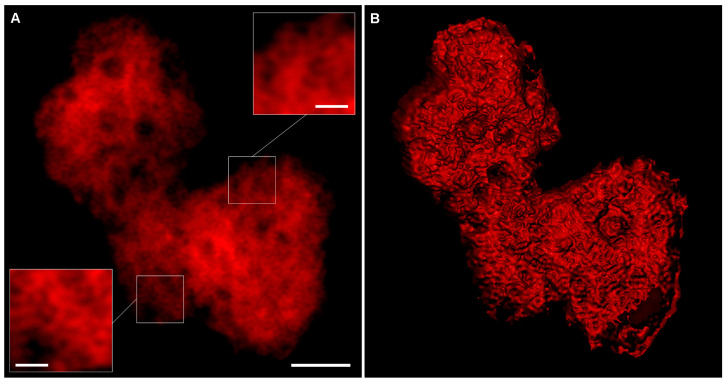
AiryScan confocal imaging of PAMB in a cell expressing mRFP-fused BMB2. (**A**) Single optical section. Insets show magnified regions of the image with higher brightness. (**B**) Three-dimensional reconstruction based on six consecutive optical sections. Scale bar, 2 μm. Scale bars for insets, 500 nm.

Transient expression of HGSV BMB2 in plant cells is sufficient to induce the formation of PAMBs [23]. Therefore, to analyze the structure of PAMBs, *Nicotiana benthamiana* leaves were agroinfiltrated for expression of BMB2-mRFP, then BMB-specific PAMBs in cells of infiltrated leaf areas were imaged by conventional confocal laser scanning microscopy at 3 days post-infiltration (dpi). As expected, PAMBs were observed in BMB2-mRFP expressing cells (Supplementary Figure S1). To gain further insight into PAMB structure, higher resolution AiryScan confocal imaging was used. Images obtained by processing primary microscopy data demonstrated a reticulate pattern of BMB2 localization in PAMBs (Figure 1A,B). As BMB2 is associated with ER membranes [23], the internal reticulate substructures observed in PAMBs by AiryScan microscopy may represent modified ER tubules; however, the resolution of this microscopy technique was apparently insufficient to determine fine details of PAMB structure.

### 2.2. Analysis of the Structure of PAMBs by Transmission Electron Microscopy

**Figure 2:**
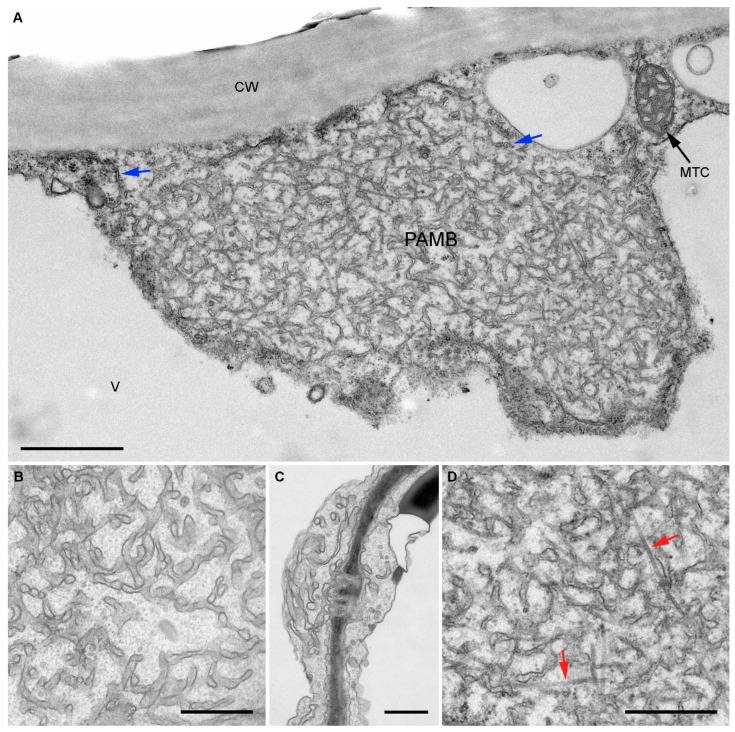
Transmission electron microscopy of PAMBs. (**A**) General view of a cell-wall-adjacent PAMB. CW, cell wall; V, vacuole; MTC, mitochondrion. (**B**) Modified ER tubules in PAMB. (**C**) Link of the PAMB ER tubules to desmotubules in PD. (**D**) Microtubules in PAMB. A magnified region of (**A**) is shown. Blue arrows point to the granular ER; red arrows point to microtubules. Scale bars, 1 μm in (**A**), 500 nm in (**B**–**D**).

The fine structure of BMB2-specific PAMBs was examined by transmission electron microscopy (TEM). Samples of leaves infiltrated with agrobacteria for expression of BMB2 were collected at 3 dpi and processed for TEM. Analysis of plant tissue sections revealed that in samples of control leaves, which have been infiltrated with agrobacteria carrying an empty vector, a thin layer of the cortical cytoplasm contained a few individual tubules of granular and smooth ER (Supplementary Figure S2). On the contrary, the peripheral cytoplasm in BMB2-expressing cells had expanded regions containing PAMBs formed by a network of ER-derived structures visible as tubules on thin sections (Figure 2A). Based on measurements made on TEM images, the average width of these PAMB membrane structures was 44.95 ± 19.3 nm, showing no statistically significant difference from the average width of the granular ER tubules used as a control, which was determined to be 37.6 ± 10.3 nm (Supplementary Figure S3). The latter value is close to the average diameter of ER tubules in *Arabidopsis* cells, reported to be 40.5 ± 0.8 nm [44]. Analysis of the width distribution revealed a higher variation in the width of the PAMB membrane structures compared to that of the ER tubules, with the proportion of wider structures most obviously increased (Supplementary Figure S3). Therefore, the TEM data indicate that the ER-derived membrane structures in BMB2-induced PAMBs are generally similar in width to the ER tubules. 

It should be noted that the membrane structures within PAMBs were located at variable distances from each other and branched without forming ordered structures (Figure 2A,B). No or few ribosomes were found on the ER tubules within PAMBs, but the periphery of PAMBs was found to border with and possibly connect to the granular ER (Figure 2A). In addition, the modified ER tubules of PAMBs were found to be connected to desmotubules of PD (Figure 2C). Occasionally, microtubules (MTs) were found within PAMBs (Figure 2D). As the positions of MTs were not ordered or regular, and MTs were not found in all examined PAMBs, it is unlikely that the MTs could form a backbone for the formation of PAMBs. Interestingly, in all analyzed PAMBs, organelles present in the areas of peripheral cytoplasm containing PAMBs were located at the periphery of these areas and were never found within the PAMBs (Figure 2A), suggesting that PAMBs may be unified, non-penetrable structures. Therefore, the TEM data indicate that the ultrastructure of BMB2-induced PAMBs is generally reminiscent of that observed by high-resolution light microscopy for replication/movement-associated membrane bodies formed upon expression of TGB proteins [32,33]. 

### 2.3. Analysis of the Structure of PAMBs by Electron Tomography

**Figure 3:**
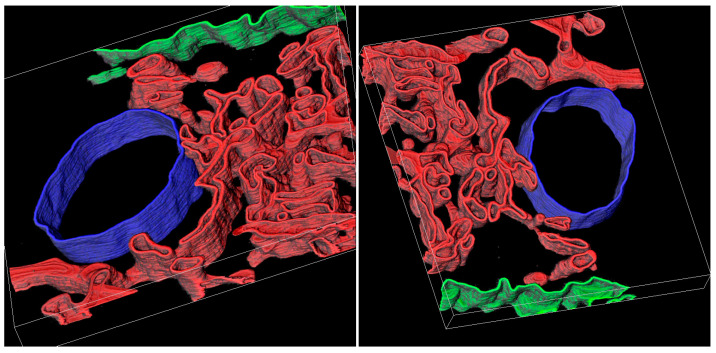
Three-dimensional reconstruction of a PAMB fragment based on tomography data. Two views of the obtained 3D reconstruction are shown. The colors are as follows: red color indicates derivatives of the ER membranes, green color indicates the plasma membrane, and blue color indicates a vacuole. The size of the model is 1500 × 1300 × 150 nm.

To further investigate the internal organization of PAMBs, we employed dual-axis EM tomography on 200 nm thick epoxy-embedded sections with gold fiducial markers. The resulting tomogram has a pixel size of 0.97 nm in the XY-plane and a section thickness of 1.87 nm. To assess the spatial arrangement of the PAMB membranes, we built a 3D model based on the tomography data by tracing the PAMB membranes, the plasma membrane, and the associated unidentified vesicle. The model revealed the variability of membrane shapes within PAMBs, ranging from smooth circular tubular to distorted swollen compartments (Figure 3). The ER-derived membranes were visible as a convoluted membrane system that included tubular, sheet-like, and distorted compartments of widely varying dimensions, forming a continuous network (Figure 3). In addition, isolated membrane vesicles were occasionally found in PAMBs (Supplementary Figure S4), suggesting that not all membrane structures in PAMBs are unified in the network. Importantly, the 3D reconstruction clearly shows that tubules are a minority among the PAMB ER-derived structures, with most of them being cisterns of different sizes (Figure 3). Therefore, most of the PAMB-specific membrane structures visible as tubules in the TEM images (see above) most likely correspond to cross-sections of ER-derived cisterns.

**Figure 4:**
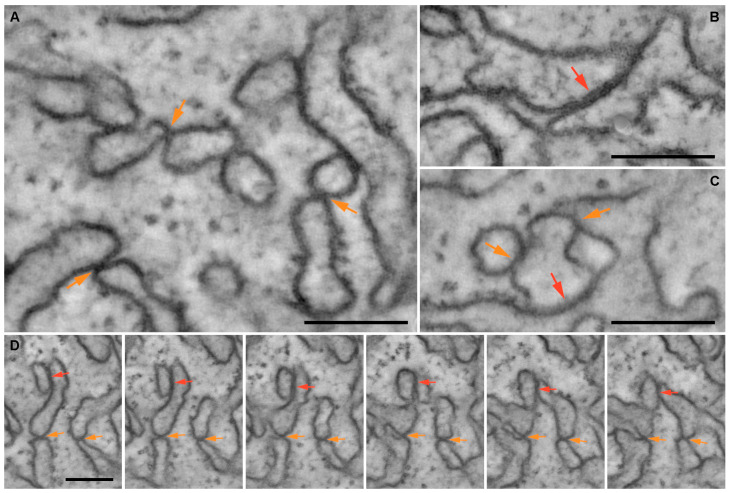
EM tomography images of intermembrane contacts in PAMBs. (**A**–**C**) Diversity of intermembrane contacts in PAMBs. (**D**) A series of PAMB sections made at an interval of 18.8 nm showing intermembrane contacts maintained over a stack of consecutive sections. Orange and red arrows indicate “point” and extended intermembrane contacts, respectively. Scale bar, 150 nm.

A closer examination of individual electron tomography images of PAMBs revealed that in many places, the membranes of neighboring ER-derived structures were locally brought closer together, forming intermembrane contacts in which no visible gap between two contacting membranes could be detected (Figure 4A). Morphologically, the intermembrane contacts in PAMBs fall into two classes. As can be seen from individual tomography images, one class includes very local, or “point”, intermembrane contacts (Figure 4A), while the other class includes extended contacts that can be as long as 200 nm (Figure 4B). In many cases, PAMB-specific membranes are locally interconnected by complex interactions involving both “point” and extended contacts (Figure 4C). Analysis of a series of tomographic sections revealed that intermembrane contacts visible on single sections as “point” contacts between the ER cisterns can be maintained over a stack of consecutive sections and thus be at least 190 nm long (Figure 4D). Therefore, the “point” contacts are often just cross-sections of extended contacts. These observations suggest that in the complex three-dimensional structure of PAMBs, there is no clear distinction between “point” and extensive intermembrane contacts. This conclusion is further illustrated by the observation that an extended contact can be transformed into a “point” contact in a series of sections (Figure 4D).

Further classification of intermembrane contacts in PAMB electron tomography images was based on the differentiation of contacts according to the degree of curvature of the membranes that form them. More specifically, three types of intermembrane contacts were distinguished: (1) contacts between two curved cisternal rims, (2) contacts between a cisternal rim and a membrane with low curvature, and (3) contacts between two membranes with low curvature. Of 438 contacts examined on EM tomography images, 50.9% were type 1 contacts between two curved cistern rims, whereas 77.4% were contacts involving one or two curved cisternal rims (types 1 and 2). Therefore, the majority of intermembrane contacts in PAMBs involve curved cistern rims. Taken together, the tomography analyses demonstrate that PAMBs are formed by a complex network of ER-derived tubules and cisterns connected by intermembrane contacts. However, the exact structure and molecular composition of these contacts await further experimental characterization.

### 2.4. Analysis of BMB1 and BMB2 Localization in PAMBs and Plasmodesmata

**Figure 5:**
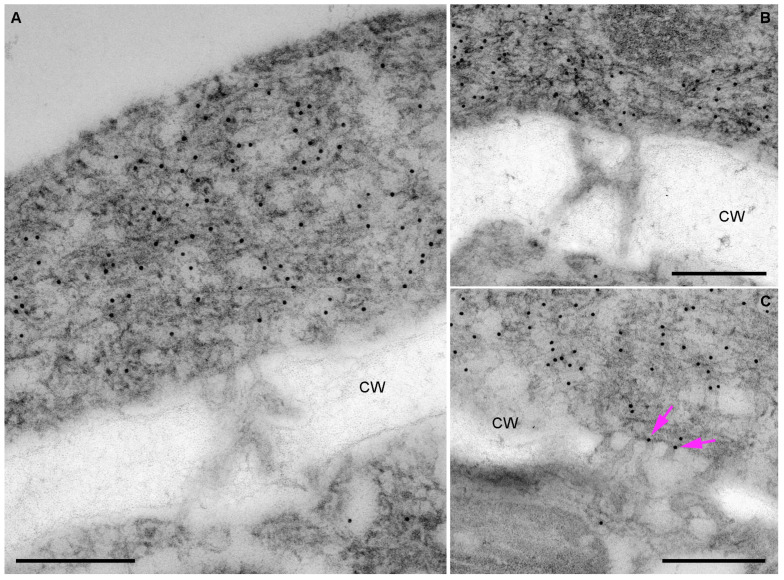
Detection of mRFP-fused BMB2 by immunogold labeling. (**A**,**B**) Localization of the fusion protein in PAMB. (**C**) Localization of the fusion protein at the PD orifice is indicated by pink arrows. CW, cell wall. mRFP antibodies were used to detect the fusion protein. Scale bars, 300 nm.

To determine the intracellular localization of the two HGSV MPs in PAMBs, immunogold labeling was used. In one series of experiments, *N. benthamiana* plants were agroinfiltrated for expression of mRFP-fused BMB2. Samples of agroinfiltrated leaves were collected at 3 dpi and processed for immunogold labeling. Detection of the fusion protein was carried out with mRFP-specific antibodies and secondary antibodies conjugated to 12 nm colloidal gold. Analysis of BMB2-expressing cells revealed that BMB2-specific labeling was strongly associated with PAMBs, with the gold label being distributed uniformly within PAMBs and showing negligible occasional association with other cell parts (Figure 5A,B). Inspection of PD in cell wall regions adjacent to PAMBs failed to detect immunogold labeling inside the PD channels (Figure 5A,B). However, in some instances, gold particles were detected at the PD orifice (Figure 5C). 

**Figure 6:**
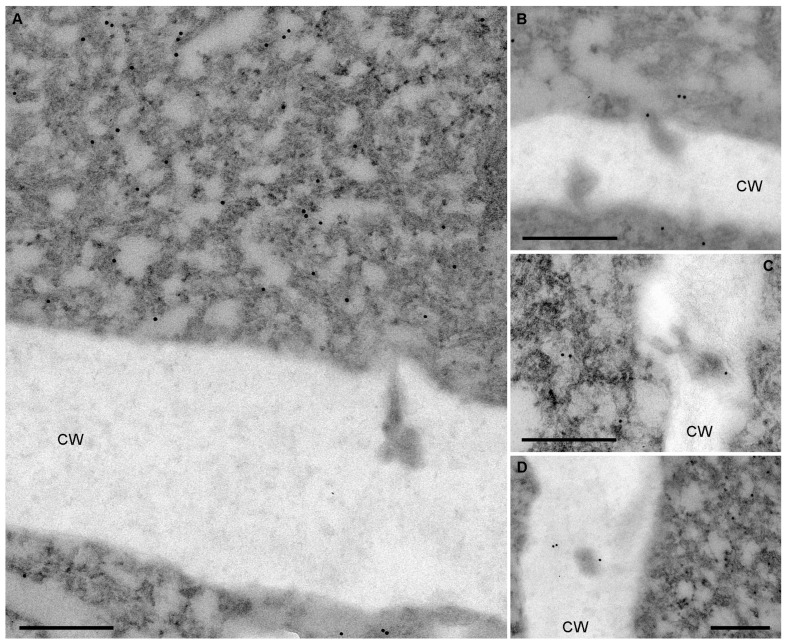
Immunogold labeling of GFP-BMB1 in cells coexpressing GFP-BMB1 and BMB2. (**A**) Localization of GFP-BMB1 in PAMBs. (**B**) Localization of GFP-BMB1 at the PD orifice. (**C**,**D**) Gold labeling in the cell wall likely associated with the PD channels. GFP antibodies were used to detect the fusion protein. CW, cell wall. Scale bars, 300 nm.

BMB1 has been shown to be targeted by BMB2 to BMB2-containing PAMBs [23]. Therefore, in another series of experiments to analyze BMB1 localization, *N. benthamiana* leaves were agroinfiltrated for coexpression of GFP-BMB1 and BMB2. It should be noted that GFP-BMB1 has been shown to be fully functional in virus cell-to-cell movement [23]. Samples collected at 3 dpi were subjected to immunogold labeling for BMB2 detection, with the exception of the use of GFP-specific antibodies. Similar to BMB2, GFP-BMB1-specific labeling was strongly associated with PAMBs and distributed throughout these structures (Figure 6A), consistent with the recently demonstrated interaction between BMB1 and BMB2 in PAMBs [39]. Analysis of cell wall regions adjacent to PAMBs revealed that immunogold labeling was typically not associated with the PD channels (Figure 6A) and was occasionally found at the PD entrance (Figure 6B). In addition, the label was associated with 2 of 18 PD examined (Figure 6C,D), consistent with previous data showing colocalization of GFP-BMB1 coexpressed with BMB2 with PD-associated callose [23]. However, the observed low efficiency of the PD interior labeling in BMB1 immunogold detection experiments may indicate that the method used to process tissue sections was not fully compatible with the labeling of internal PD structures, which may be poorly preserved under these conditions. Thus, immunogold detection of BMB1 and BMB2 generally confirms observations previously made using confocal microscopy [23]; however, definitive conclusions regarding the localization of both proteins in the PD channels could not be drawn from these data.

### 2.5. BMB2 Self-Interaction in PAMBs

**Figure 7:**
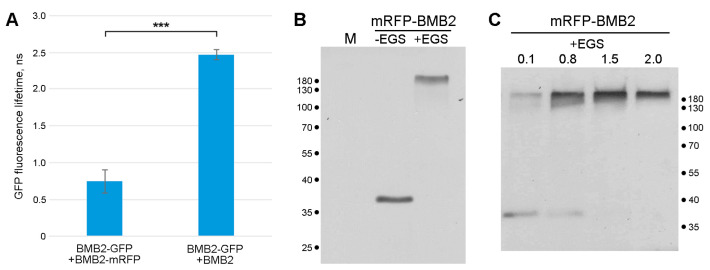
BMB2 interactions in PAMBs. (**A**) FRET-FLIM analysis of BMB2 self-interaction in cells of *N. benthamiana* leaves agroinfiltrated for expression of BMB2-GFP and either BMB2-mRFP or BMB2. Average fluorescence lifetimes (ns) are shown; error bars indicate the standard deviation. Asterisks indicate a statistically significant difference (*p* < 0.001) according to a Student’s *t*-test. (**B**) Cross-linking analysis of protein complexes formed by BMB2. Cell membrane fraction from leaves agroinfiltrated for expression of mRFP-fused BMB2 was treated with EGS (2 mM) and analyzed by Western blotting together with a non-treated sample. M, a sample from non-infiltrated leaf. (**C**) EGS cross-linking performed at four increasing EGS concentrations as indicated above the lanes (mM). The positions of protein molecular weight markers are indicated in kDa.

As BMB2 is, at least in part, functionally similar to reticulons [24], and the latter are known to oligomerize in the ER membrane to generate the curvature of the lipid bilayer [43], the potential of BMB2 for self-interaction was investigated. For this purpose, FRET-FLIM (Förster resonance energy transfer between two fluorophores detected by fluorescence-lifetime imaging microscopy) was used. In this approach, the excited-state lifetime of GFP (a donor fluorophore) is measured in the presence and absence of mRFP (an acceptor fluorophore). When the distance between the two fluorophores is less than 10 nm, energy transfer from GFP to mRFP occurs, resulting in a reduced excited-state lifetime of GFP, indicative of interaction between GFP- and mRFP-fused proteins [45]. Therefore, leaves of *N. benthamiana* plants were agroinfiltrated for the coexpression of BMB2-GFP (donor) with either BMB2-mRFP (acceptor) or non-fused BMB2 (a control without acceptor), and the GFP fluorescence lifetime was measured in PAMBs at 3 dpi. The GFP excited-state lifetime measured for the coexpression of BMB2-GFP with BMB2-mRFP (0.74 ± 0.16 ns; N = 78) was found to be significantly lower than that for the coexpression of BMB2-GFP with BMB2 (2.47 ± 0.07 ns; N = 78) (Figure 7A), indicating a FRET efficiency of 69.8%. These data show that BMB2-GFP and BMB2-mRFP interact in PAMBs, confirming the hypothesis of BMB2 self-interaction. 

To provide biochemical evidence for BMB2 interaction in vivo, a cross-linking approach was used. Leaves of *N. benthamiana* plants were agroinfiltrated for expression of BMB2 fused to mRFP; then, a cell membrane fraction was isolated from the agroinfiltrated tissue and treated with 2 mM ethylene glycol-bis(succinimidylsuccinate) (EGS), a bifunctional cross-linker containing two amine-reactive groups separated by a 12-atom spacer. Immunoblotting with mRFP-specific antibodies revealed that in non-treated samples, mRFP-fused BMB2 was detected as a single band with the mobility expected for monomeric protein, whereas in EGS-treated samples, the fusion protein appeared as a band of more than 180 kDa (Figure 7B). These data suggest that BMB2 forms high-molecular-weight complexes, likely due to the ability of BMB2 to self-interact, which may represent protein oligomers. To determine whether BMB2 dimers or other intermediate-sized complexes could be detected, the membrane fraction was treated with increasing concentrations of EGS. As shown by Western blotting, no bands between the monomeric protein and the 180 kDa complex could be detected in the gel (Figure 7C). This observation distinguishes BMB2 from reticulons, which form a “ladder” of cross-linked bands corresponding to dimers and oligomers under similar EGS treatment conditions [43].

## 3. Discussion

The data presented in this paper demonstrate that BMB2-induced PAMBs have a complex internal structure. Previous studies have shown that (1) PAMBs are composed of ER-derived membranes containing BMB2, and (2) BMB2 can induce constrictions of cortical ER tubules [23,24,46]. Based on these data, one might expect that PAMBs can be formed from constricted ER tubules. However, as shown by quantification of TEM data obtained for PAMB thin sections, no constricted membrane structures can be detected in PAMBs, and the average width of PAMB membrane structures is similar to that of ER tubules. Furthermore, EM tomography data show that tubules are a minority of PAMB membrane structures, whereas the majority are cisterns, suggesting that tubule-like structures visible on thin sections examined by TEM are mostly cross-sections of PAMB-specific cisterns. Therefore, the ability of BMB2 to induce ER tubule constrictions is not manifested when the protein is localized in PAMB-specific membrane structures. This can be explained by the cisternal rather than tubular PAMB organization and a specific relative spatial positioning of the ER cisterns in PAMBs (see below). On the other hand, as the HGSV BMB2-induced PAMBs, which are presumed to be structurally and functionally similar to PVX VRCs associated with PD [32,33], can serve as VRCs in virus infection, one can hypothesize that the observed conversion of the ER tubules into cisterns rather than constricted tubules can increase the surface of PAMB membranes required for efficient virus replication that occurs in association with membranes. It should be also noted that, as revealed by TEM, the PAMB-specific ER membranes are connected both to the PD desmotubules in the adjacent cell walls and to the granular ER in the cortical cytoplasm, thus confirming the previously postulated continuity of the ER membrane in the ER network, PAMBs and desmotubules [23,24]. 

The EM tomography data demonstrate that the ER-derived membrane structures in PAMBs are interconnected by numerous intermembrane contacts, where two membranes come very close to each other but do not coalesce. These intermembrane contacts resemble membrane contact sites (MCSs), highly specialized structures where the ER membrane interacts with the membranes of other organelles, including Golgi, vacuoles, peroxisomes, endosomes, the plasma membrane, mitochondria, and plastids, by means of MCS-specific proteins locally tethering two membranes [47]. However, to our knowledge, MCSs between ER tubules have never been reported, suggesting that the appearance of such structures in PAMBs may result from BMB2 expression. Interestingly, there is no visible distance between two membranes in the intermembrane contacts in PAMBs, whereas typical MCSs between the ER and other organelles are characterized by an intermembrane gap of up to 15–20 nm [47], suggesting a structural difference between the PAMB-specific intermembrane contacts and most known MCSs. Therefore, further studies are required to determine whether the intermembrane contacts in PAMB are related to archetypal MCSs and contain MCS-specific proteins. Regardless of the exact nature of the intermembrane contacts in PAMBs, it should be emphasized that PAMBs contain numerous such contacts that form a 3D interaction network, and therefore, the ER cisterns in PAMBs appear to be tightly interconnected by these contacts. Based on these considerations, we hypothesize that intermembrane contacts hold the PAMB structure together. 

Chemical cross-linking experiments show that BMB2 forms complexes of more than 180 kDa in plant cells. Their composition remains undetermined; however, taking into account the strong ability of BMB2 to self-interact demonstrated by FRET-FLIM, these complexes can be expected to contain dimers and/or oligomers of BMB2. As no BMB2 complexes of sizes between the protein monomer and the 180 kDa complex are detected even at low concentrations of the cross-linking reagent, the formation of BMB2-containing complexes may be a highly cooperative process. These data suggest that the formation of complexes containing protein multimers is different for BMB2 and reticulons, as the latter proteins form readily detectable dimers and oligomers [43], suggesting low cooperativity of interaction between reticulon molecules. It should be noted that cell proteins, speculatively those that interact with BMB2, may be part of the observed BMB2-specific complexes.

Analysis of the EM tomography data indicates that the interaction of PAMB membrane cisterns that results in the formation of intermembrane contacts predominantly involves the rims of cisterns rather than their flat surfaces. Previously, we have shown that in plant cells, where the network of cortical ER tubules is artificially converted into cisterns, BMB2 is located at the cistern rims, showing an affinity for membrane regions with high curvature [24]. Therefore, it can be speculated that BMB2 is located at the rims of ER-derived cisterns in PAMBs. In this case, the interaction of BMB2 molecules located on different cisterns may lead to the formation of intermembrane contacts, implying that the intermembrane contacts found in PAMBs can be formed without proteins typical for MCSs. To verify this model, the ability of membrane-integrated BMB2 molecules to homotypically interact with BMB2 embedded in another membrane should be determined experimentally.

Taking into account previously published data on BMB2 interaction with ER membranes and the new data reported here, we hypothesize that BMB2-dependent formation of PAMBs may occur in two steps. Initially, integration of BMB2 into the ER membrane can induce ER tubule constrictions. Next, due to the interaction of BMB2 molecules, BMB2-modified ER tubules can be interconnected by BMB2-BMB2 junctions and reorganized into cisterns, where BMB2 is displaced from flat side membranes to cistern rims with high membrane curvature. It cannot be excluded that cell proteins are also involved in the BMB2-induced reorganization of ER tubules. Such involvement is known for animal picornaviruses, which manipulate cell proteins such as factors of membrane remodeling and lipid biosynthesis to induce VRC formation [48].

The molecular mechanism of virus cell-to-cell transport mediated by BMB proteins is not fully understood. Previous studies based on confocal microscopy have shown that BMB2 can direct BMB1 to the BMB2-containing PAMBs, to the PD interior, and to neighboring cells through the PD channels [23]. Immunogold labeling data confirm the localization of both BMB1 and BMB2 in PAMBs, showing the association of both proteins with PAMB membranes and the PD orifices. In the case of BMB2, these experiments could not verify the model of BMB2 localization at the cisternal rims, as the membranes are generally not well preserved during the section treatments required for immunogold microscopy, and the spatial localization of BMB2 in PAMBs is difficult to resolve by thin section analysis. It should be noted that BMB1, but not BMB2, is found in the PD channels, consistent with previously reported confocal microscopy observations showing colocalization of BMB1 with PD-associated callose [23]. However, as only 11% of PD examined by immunogold microscopy contain the label in the PD channels, these observations cannot confidently confirm the confocal microscopy data. We assume that the efficient detection of BMB1 in the PD channels could be precluded by the limitations of the methods used. For example, the internal structure of the PD channels may be poorly preserved during sectioning and/or subsequent processing of the tissue samples. Nevertheless, the limited immunogold labeling data are consistent with the model postulating that BMB1 can be transported from PAMBs to neighboring cells.

Taken together, the data on the fine structure of BMB2-induced PAMBs provide new insights into the reorganization of cell membranes by viral proteins. Moreover, the novel data on the predominantly cisternal organization of membranes in PAMBs and the presence of intermembrane contacts in PAMBs open new directions for future investigations of the mechanism of BMB-mediated virus cell-to-cell transport, such as characterization of the molecular composition of PAMB-specific intermembrane contacts and analysis of the mechanism of cortical ER tubules conversion into cisternal structures.

## 4. Materials and Methods

### 4.1. Agroinfiltration of Plants

Recombinant constructs for the expression of BMB proteins in plants have been described previously [23]. Agrobacterial cultures were grown overnight at 28 °C in Luria–Bertani medium (Helicon, Moscow, Russia) supplemented with 10 mM 2-(N-morpholino)ethanesulfonic acid (MES, pH 5.5), 20 mM acetosyringone, and selective antibiotics. Agrobacterial cells were pelleted by centrifugation, resuspended in infiltration buffer (10 mM MES, pH 5.5, 10 mM MgCl_2_, 150 mM acetosyringone), and incubated at room temperature for 3–4 h. Prior to infiltration of plant leaves, the cell suspensions were diluted to a final optical density of 0.3 at 600 nm (OD_600_), then the agrobacterial suspensions were infiltrated into the abaxial side of young fully expanded leaves using a 2 mL syringe without a needle.

### 4.2. AiryScan Confocal Microscopy

AiryScan imaging was performed with an LSM 900 confocal laser scanning microscope (Carl Zeiss AG, Oberkochen, Germany) equipped with a 63× oil-immersion objective and an AiryScan detection unit. ZEN Blue 3.4 software was used for Wiener filter deconvolution and reconstruction of acquired images.

### 4.3. Sample Collection and Preparation for EM

For the ultrastructural studies, approximately 5 × 1 mm fragments were excised from *N. benthamiana* leaves and vacuum-infiltrated with 2.5% glutaraldehyde (GA) solution in 100 mM sodium cacodylate. The samples were fixed in GA for 24 h, rinsed three times for 5 min in 100 mM sodium cacodylate, and postfixed in 1% osmium tetroxide in 100 mM sodium cacodylate for 1 h at +4 °C. Samples were dehydrated through increasing concentrations of ethanol (50%–70%–96%). Subsequently, 96% ethanol was replaced with acetone, followed by epoxy resin–acetone mixtures with increasing resin content. After replacing the mixture with pure resin Spi-pon 812 (SPI Supplies, West Chester, PA, USA), the resin was cured at +70 °C for 48 h.

For EM immunocytochemistry, leaf fragments were similarly fixed with 1% GA for 60 min at +4 °C, washed with cold 100 mM sodium cacodylate, and transferred into 40% ethanol for 20 min, followed by 50% and 70% ethanol for 30 min each. Samples were pre-infiltrated by immersion in a 5:1 mixture of LR White resin and 70% ethanol for 1 h and infiltrated overnight at +4 °C with two changes of fresh LR White. Samples were then transferred to the silicone rubber embedding molds filled with fresh LR White. The molds were placed in a sealed glass container, and the air in the vessel was replaced with CO_2_. LR White was then allowed to cure at +55 °C for 48 h.

Ultrathin sections with a nominal thickness of 70 nm and semithin sections (200 nm) for EM tomography were prepared using a Reichert-Jung Ultracut E ultramicrotome equipped with an Ultra 45 diamond knife (Diatome, Nidau, Switzerland).

For morphological studies and EM tomography, sections were mounted on formvar-coated copper slot grids and post-stained with 1% aqueous uranyl acetate for 1 h and with lead citrate for 90–120 s.

For EM tomography, after post-staining, the sections were coated with poly-L-lysine, and the colloidal gold particles were applied as described previously [49]. The sections were then carbon-coated using a HUS-3B vacuum evaporator (Hitachi, Tokyo, Japan).

For immunocytochemistry, the LR White sections were mounted on formvar-coated gold slot grids and processed immediately.

### 4.4. EM Immunocytochemistry

The LR White sections on formvar-coated gold slot grids were etched with 10% hydrogen peroxide for 10 min, washed thoroughly with three changes of PBS, and blocked in 3% BSA in PBS for 60 min. Rabbit antibodies against GFP and RFP (Evrogen, Moscow, Russia) were diluted 1:100 and 1:30, respectively, in PBS supplemented with 0.1% BSA. Sections were incubated in the antibody solution at +4 °C overnight. The sections were washed three times for 10 min in antibody dilution buffer and incubated with 12 nm colloidal gold-conjugated goat anti-rabbit antibodies (Jackson ImmunoResearch, Ely, UK) diluted 1:100 in the same buffer for 1 h at room temperature. Excess antibodies were removed by washing the sections once in dilution solution, three times in PBS, and twice in distilled water. The sections were then air-dried. Prior to observations, the sections were poststained with 1% aqueous uranyl acetate for 1 h and with lead citrate for 90–120 s. Each experiment was repeated twice; at least ten individual sections were examined in each repetition.

### 4.5. Transmission Electron Microscopy and Tomography

Sections were observed and photographed with a JEM-1400 electron microscope (JEOL, Tokyo, Japan) operating at 80 kV and equipped with a Quemesa digital camera (Olympus Scientific Imaging Solutions, Munster, Germany). The experiment was repeated twice; at least ten individual sections were examined in each repetition.

EM tomography studies were performed with a JEM-2100 200 kV electron microscope (JEOL) equipped with a LaB_6_ cathode. Three adjacent ultrathin sections were used for tomography data acquisition. Images were captured with US1000FTXP CCD (Gatan, Pleasanton, CA, USA) at 0.97 nm image pixel size and −2 µm defocus. A GIF electron energy filter (Gatan) with a 20 eV zero-loss slit was used to filter out inelastically scattered electrons. Dual-axis tomograms were acquired using SerialEM (v.3.8.16) [50] with a tilt range of −60 to +60 deg and a tilt step of 1 deg. The collected data were processed using IMOD (v.4.11.0) [51]. Gold fiducials were used for both image alignment and merging of tomograms acquired from different tilt axes and ultrathin sections. Reconstruction was performed with a filtered back-projection algorithm using the SIRT-like filter. Three EM tomography datasets were obtained; two datasets were used for 3D reconstruction; one reconstruction was presented.

### 4.6. EM Image Processing and Measurements

Segmentation of tomographic images was performed by manual tracing of the membrane contours in GIMP using a pen tablet. The 3D model based on the obtained contours was assembled and visualized using the Volume Viewer plug-in for FIJI (version 2.14.0). Endoplasmic reticulum width measurements were performed in FIJI. Images were calibrated using microtubules as a reference. Membrane cisterns were measured from one outer edge to the other edge of the structure.

### 4.7. FRET-FLIM

FRET-FLIM measurements were performed using a LIFA frequency-domain fluorescence-lifetime imaging system (Lambert Instruments, Roden, The Netherlands) and with a DCS-120 TCSPC confocal FLIM system (Becker and Hickl, Berlin, Germany). Independent experiments were repeated at least three times, with at least three leaves used for each pair of coexpressed proteins in each experiment. Raw data were analyzed in Microsoft Excel. A two-tailed parametric Student’s *t*-test was used for statistical analysis. FRET efficiency was calculated using the formula E = 1 − (T_DA_/T_D_), where T_DA_ is the lifetime of the donor fluorophore (GFP) in the presence of the acceptor fluorophore (mRFP), and T_D_ is the lifetime of the donor in the absence of the acceptor.

### 4.8. Isolation of a Membrane Fraction and Chemical Cross-Linking Experiments

The membrane fraction was isolated as described previously [52]. *N. benthamiana* leaves were ground to a fine powder in liquid nitrogen and resuspended in high-density extraction buffer (EB; 100 mM Hepes, pH 7.5, 25% (*w*/*w*) sucrose, 5% (*v*/*v*) glycerol, 10 mM EDTA, 5 mM KCl, 1 mM DTT, 1 mM PMSF). A total of 1 mL EB was used per 1 mg of plant material. Crude homogenates were precleared at 600 g and then at 1500 g. Total membrane fractions were pelleted in a final centrifugation step at 21,000× *g*. The membrane pellets were washed with wash buffer (50 mM Hepes, pH 7.5, 5 mM EDTA, and 1 mM PMSF) and recentrifuged at 21,000× *g*. All steps were carried out on ice and in a cooled (4 °C) microcentrifuge. The membrane pellets were then resuspended in reaction buffer (100 mM Hepes, pH 7.5, 5% (*v*/*v*) glycerol, 10 mM EDTA, 5 mM KCl, 1 mM PMSF).

Ethylene glycol-bis(succinimidylsuccinate) (Merck, Darmstadt, Germany) was dissolved in DMSO to a stock concentration of 30 mM. Isolated membranes were treated with EGS for 60 min at room temperature. The reactions were then quenched by adding 1 M Tris-HCl, pH = 7.5, to a final concentration of 20 mM and incubated for 30 min. Samples were analyzed by 10% SDS-PAGE, and complexes were visualized by standard immunoblotting procedures with anti-tRFP antibodies (Evrogen, Moscow, Russia). 

## Data Availability

Data are contained within the article and Supplementary Materials.

## Supplementary Materials

The following supporting information can be downloaded at https://www.mdpi.com/article/10.3390/plants12244100/s1, Figure S1: PAMBs in *N. benthamiana* cells expressing BMB2-mRFP; Figure S2: Cortical cytoplasm in cells of a leaf agroinfiltrated with empty vector (a negative control); Figure S3: Analysis of the width of membrane structures in PAMBs based on transmission electron microscopy data; Figure S4: Vesicles revealed in PAMB by electron tomography.
