# Supplementary material for: Fine Structure of Plasmodesmata-Associated Membrane Bodies Formed by Viral Movement Protein
Source: Plants (Basel). 2023 Dec 7;12(24):4100. doi: 10.3390/plants12244100 (PMC10747570; doi:10.3390/plants12244100)
Supplement: Supplementary file 1 [file plants-12-04100-s001.zip › plants-2715673-supplementary.pdf]

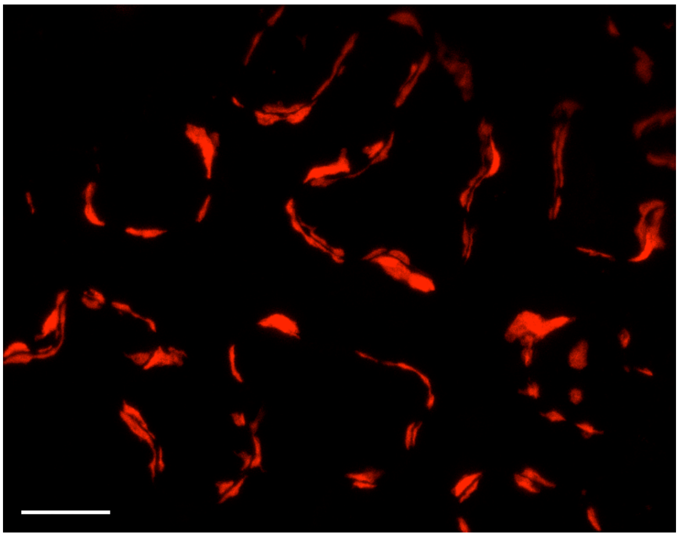

Figure S1. PAMBs in *N. benthamiana* cell expressing BMB2-mRFP. The image is assembled from a z-stack of optical sections obtained by confocal laser scanning microscopy. Scale bar, 20 μm.

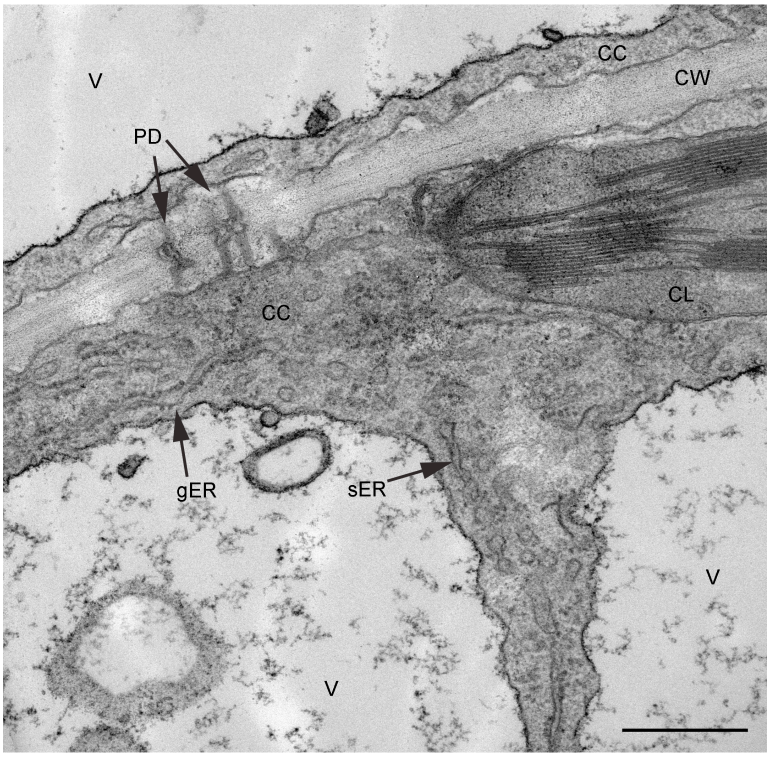

Figure S2. Cortical cytoplasm in cells of a leaf agroinfiltrated with empty vector (a negative control). CW, cell wall; CC, cortical cytoplasm; V, vacuole; CL, chloroplast; PD, plasmodesmata; gER, granular ER; sER, smooth ER. Scale bar, 500 nm.

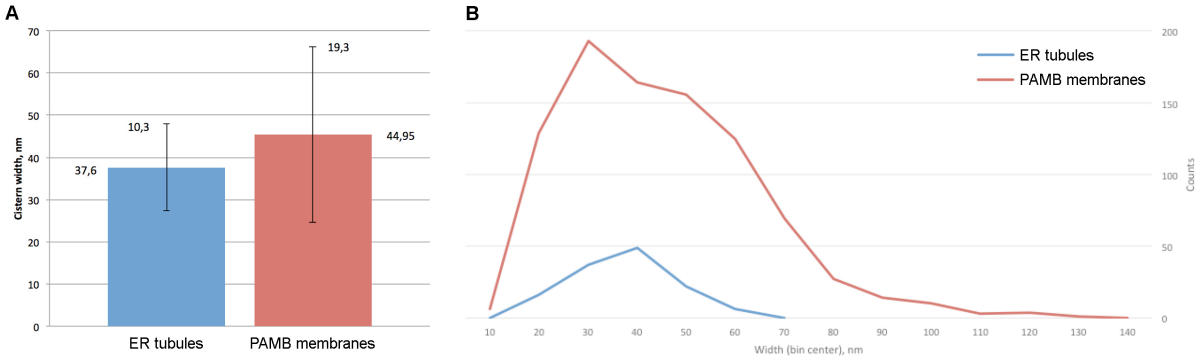

Figure S3. Analysis of the width of membrane structures in PAMBs based on transmission electron microscopy data. (**A**) Bar graphs show the average width of PAMB membrane structures compared to the average width of tubules of the granular ER. Error bars indicate the standard deviation. (**B**) Plot of width distribution in membrane structures showing the number of measurements plotted for 10-nm bins of measured width. The number of independent measurements (N) was 901 for PAMB membranes and 130 for ER tubules.

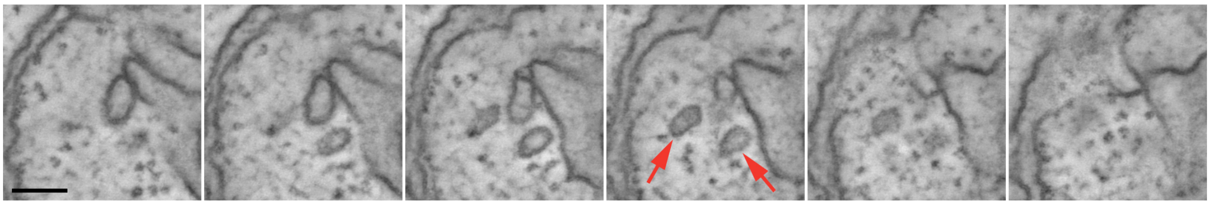

Figure S4. Vesicles revealed in PAMB by electron tomography. PAMB sections made at 18.8 nm intervals are shown. Red arrows indicate two irregularly shaped vesicles that do not contact surrounding membrane structures in any of the consecutive images. Scale bar, 100 nm.
